# Mitochondria-Mediated Cardiovascular Benefits of Sodium-Glucose Co-Transporter 2 Inhibitors

**DOI:** 10.3390/ijms23105371

**Published:** 2022-05-11

**Authors:** Siarhei A. Dabravolski, Alexander D. Zhuravlev, Andrey G. Kartuesov, Evgeny E. Borisov, Vasily N. Sukhorukov, Alexander N. Orekhov

**Affiliations:** 1 Department of Clinical Diagnostics, Vitebsk State Academy of Veterinary Medicine [UO VGAVM], 7/11 Dovatora str., 210026 Vitebsk, Belarus; 2 AP Avtsyn Research Institute of Human Morphology, 3 Tsyurupa Street, 117418 Moscow, Russia; zhuravel17@yandex.ru (A.D.Z.); borisovevgenij5@gmail.com (E.E.B.); vnsukhorukov@gmail.com (V.N.S.); 3 Laboratory of Angiopathology, Institute of General Pathology and Pathophysiology, Russian Academy of Medical Sciences, 125315 Moscow, Russia; andkartuesv@gmail.com; 4 Institute for Atherosclerosis Research, Osennyaya 4-1-207, 121609 Moscow, Russia; a.h.opexob@gmail.com

**Keywords:** SGLT2 inhibitors, diabetes mellitus, mitochondria, cardiovascular diseases, empagliflozin, dapagliflozin

## Abstract

Several recent cardiovascular trials of SGLT 2 (sodium-glucose cotransporter 2) inhibitors revealed that they could reduce adverse cardiovascular events in patients with T2DM (type 2 diabetes mellitus). However, the exact molecular mechanism underlying the beneficial effects that SGLT2 inhibitors have on the cardiovascular system is still unknown. In this review, we focus on the molecular mechanisms of the mitochondria-mediated beneficial effects of SGLT2 inhibitors on the cardiovascular system. The application of SGLT2 inhibitors ameliorates mitochondrial dysfunction, dynamics, bioenergetics, and ion homeostasis and reduces the production of mitochondrial reactive oxygen species, which results in cardioprotective effects. Herein, we present a comprehensive overview of the impact of SGLT2 inhibitors on mitochondria and highlight the potential application of these medications to treat both T2DM and cardiovascular diseases.

## 1. Introduction

Type 2 diabetes mellitus (T2DM) is a growing public health problem worldwide, with a rising prevalence and a high mortality rate. Diabetes mellitus refers to a group of metabolic disorders associated with a long-term elevated blood glucose level. Diabetes mellitus is linked with complications that mainly affect the cardiovascular system, kidneys, eyes, and nervous system. Currently, approximately 537 million people worldwide are estimated to have diabetes, and this proportion is predicted to increase with time. T2DM is a more common type of diabetes and accounts for approximately 89% of diagnosed cases [1]. T2DM patients have about twice the risk of CVD (cardiovascular diseases) than those without diabetes, with atherosclerosis and heart failure as the most common complications and significant causes of morbidity and mortality in T2DM patients [2,3]. However, early control of the blood glucose levels could minimise the risk of CVD, diabetic nephropathy, and mortality [4]. Currently, many glucose-lowering drugs are used in different strategies to reduce the T2DM associated risk of mortality, major cardiovascular events, and other unwanted side effects [5,6,7].

Because the glucose concentration in plasma is under tight regulation within narrow limits (4–10 mmol/L), it was suggested that the kidneys play a crucial role in glucose homeostasis in the body by preventing glucose loss with the urine via specific transporter pumping of glucose back to the plasma. The original SGLTs (Na^+^-glucose co-transporter) concept was first proposed in 1960 by Dr Crane [8], with the first SGLT cloned only in 1987 [9]. Currently, there are six SGLT subtypes recognised, of which SGLT1 and SGLT2 are the most important. SGLT1 has a high affinity and low transport capacity for glucose, and it is expressed in the brain, heart, intestine, skeletal muscle, trachea, prostate, and kidney. On the contrary, SGLT2 has a low affinity and high transport capacity for glucose and is located almost exclusively in the epithelium of the proximal tubular segment [10]. Usually, >90% of filtered glucose is reabsorbed by SGLT2 in the proximal tubule, while SGLT1 reabsorbs the rest (<10%) in more distal segments of the proximal tubule [11].

Currently, several SGLT2 inhibitors are approved by the FDA as a new class of antihyperglycemic drugs for T2DM patients (but not T1DM) [12,13]. These drugs enhance urinary glucose excretion by inhibiting renal glucose reabsorption in the early proximal tubule, subsequently lowering the glucose burden on the organism. Several large-scale clinical trials were designed to confirm cardiovascular and microvascular safety for SGLT2 inhibitors (empagliflozin, dapagliflozin, and canagliflozin). Application of Canagliflozin in T2DM patients demonstrated a lower risk of stroke, myocardial infarction, cardiovascular death, and hospitalisation for heart failure [14]. Treatment of T2DM patients at high cardiovascular risk with empagliflozin demonstrated slower progression of kidney disease and lower rates of clinically relevant renal events [15]. In patients with chronic kidney disease, dapagliflozin has shown a lower risk of death from renal or cardiovascular causes, regardless of the presence or absence of T2DM, suggesting its therapeutic potential in the non-diabetic setting [16,17].

Further, in this review, we focus on the pharmacological and molecular mitochondria-mediated mechanisms of SGLT2 inhibitors, associated with their cardioprotective properties.

## 2. Proposed Pharmacological Mechanisms of SGLT2 Inhibitor Effects

SGLT inhibitors have a long history of investigation. The first natural SGLT inhibitor, phlorizin, was isolated in 1835 from the root bark of apple trees. However, because of the poor absorption in the gastrointestinal tract and low water solubility, phlorizin and some of its early derivatives (such as T-1095) were not suitable for clinical development as anti-hyperglycaemic agents [reviewed in [18]]. Eventually, more advanced C-aryl glycoside derivatives of phlorizin (such as dapagliflozin and canagliflozin) were developed, which have distinctive structural differences and variable selectivity to SGLT1 and SGLT2 [19]. Thus, canagliflozin, dapagliflozin, and empagliflozin directly inhibit SGLT2, which results in reduced glucose reabsorption, promoted urinary glucose excretion, negative caloric balance, and subsequent weight loss [20].

However, the exact mechanism of SGLT2 inhibitor cardioprotective effects is not known. Further, we discuss three main hypotheses, explaining the observed effects of SGLT2 inhibitors: (1) the diuretic effect, with subsequent hemodynamic unloading of the left ventricle; (2) the switch in cardiac metabolism to ketone utilisation (also known as “thrifty substrate” hypothesis; (3) the direct influence on cardiac ion homeostasis.

### 2.1. The Diuretic Hypothesis

SGLT1 and SGLT2 co-transport Na^+^ together with glucose, so their activity is closely connected to the Na^+^ gradient generated by the Na^+^/K^+^-ATPase between the tubular lumen and the cell. SGLT2 inhibitors reduced the reabsorption of glucose and Na^+^ in the proximal tubule in an insulin-independent way, directly affecting the total glucose pool. Such a specific mechanism of action implies that SGLT2 inhibitors do not increase the risk of hypoglycaemia; thus, they could be combined with other glucose-lowering drugs [21]. While the natriuresis effect induced by SGLT2 inhibitors is transient, the sustained increase in haematocrit and haemoglobin levels and the decrease in systolic (5–6 mmHg) and diastolic (1–2 mmHg) blood pressure may result from a persistent volume depletion [22]. Apparently, the diuretic effect associated with SGLT2 inhibition is the primary driver of its cardioprotective activity. Mechanically, the observed plasma volume contraction may hemodynamically unload the left ventricle, further decreasing myocardial O_2_ demand, ventricular wall tension, and filling pressures [23].

Moreover, treatment with SGLT2 inhibitors improves hard renal outcomes in T2DM by reducing filtration fraction without increasing renal vascular resistance. SGLT2 inhibitors reduce measured glomerular filtration rate and filtration fraction in T2DM via post-glomerular vasodilation [24]. Interestingly, the application of SGLT2 inhibitors does not affect serum levels of K^+^ and Ca^2+^ while marginally increasing serum magnesium levels in T2DM patients [25]. However, the clinical significance of elevated magnesium levels in T2DM patients requires further investigations.

### 2.2. The “Thrifty Substrate” Hypothesis

The human heart is a metabolically flexible organ, and it could use different substrates as energy sources to maintain a stable ATP production rate in response to alterations of workload and changes in substrate availability. Typically, 95% of the heart’s energy is produced by the mitochondrial oxidative phosphorylation system, which is powered by FA (fatty acids) (70%) and glucose (30%). FAs are the preferred substrates during prolonged fasting, whereas a shift towards glucose oxidation occurs after β-adrenergic stimulation or increased workload [26]. However, as a result of peripheral insulin resistance-mediated lipolysis dysregulation, the delivery of FAs to the heart is dramatically increased, subsequently increasing the contribution of FAs to oxidative metabolism. Recent research suggests that shifts in mitochondrial substrate preference are not essential in the pathogenesis of muscle insulin resistance [27,28].

On the contrary, high FA concentration in the blood defines myocardial FA uptake, changes in gene expression, and mitochondrial protein post-translational modification. Under such metabolic conditions, the expression of genes essential for FA oxidation is stimulated, whereas genes involved in glucose uptake and oxidation are suppressed [29]. Nuclear transcription factors PPAR-α (peroxisome proliferator-activated receptor alpha) and PPAR-γ are the central regulators of the genetic switch towards FA-based metabolism, activating peroxisomal and mitochondrial *β*-oxidation in the liver and heart [30,31]. Similarly, other members of the PPAR family are involved in cardioprotection and regulation of cardiac mitochondria metabolism. For example, activation of PPAR β/δ improves the endothelial dysfunction and reduces vascular inflammation [32], alleviates myocardial ischemia/reperfusion injury via stimulation of the antioxidant defense of the heart with preservation of mitochondrial function [33]. Administration of β-elemene was shown to reduce lipid-induced inflammation in a PPARβ-mediated way on a heart failure induced mouse model [34]. In addition, long-term exposure to high FAs shifts the diabetic heart to a metabolically inflexible state, where ATP production almost completely depends on FA supply. As a consequence of such a metabolic switch, ATP production is decreased, and ROS formation is increased, further promoting the development of cardiac dysfunction [35,36].

**Figure 1:**
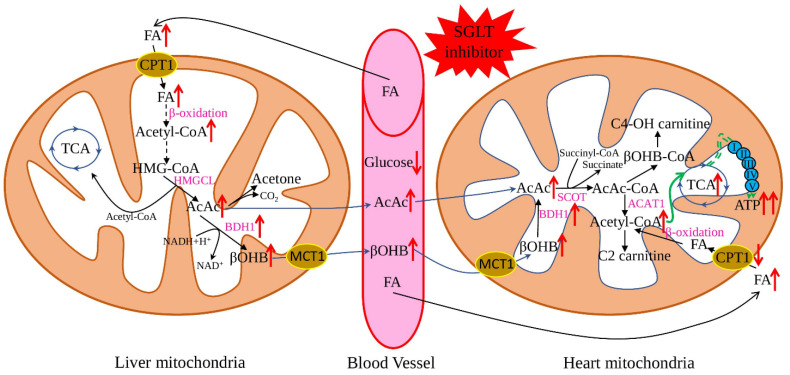
Effect of SGLT2 inhibitors on ketone body metabolism. The application of SGLT2 inhibitors reduces plasma glucose levels and subsequently promotes lipolysis in adipose tissue and FA production, which further enhances the generation of ketone bodies. Ketone bodies are converted to acetyl-CoA in the heart easier than glucose and FA. At the same time, SGLT2 inhibitors increase the expression of the key genes responsible for ketone oxidation, which leads to the metabolic shift to the ketone bodies as a preferable substrate. Red arrows show the effect of SGLT2 inhibitors on the key metabolites and enzymes; black arrows—flows of the main metabolites; green arrows—ATP production by the mitochondrial oxidative phosphorylation system. FA—fatty acids, AcAc CoA—acetoacetyl CoA, HMG-CoA—3-hydroxy-3-methtylglutaryl-CoA, βOHB—β-hydroxybutyrate, TCA—tricarboxylic acid cycle, HMGCL—3-hydroxy-3-methylglutaryl-coenzyme A lyase, BDH1—mitochondrial β-hydroxybutyrate dehydrogenase, ATP—adenosine triphosphate, ACAT1—acetyl-CoA acetyltransferase, C2-carnitine—acetylcarnitine, C4-OH carnitine—hydroxybutyrylcarnitine, CPT1—carnitine palmitoyltransferase 1, SCOT—succinyl-CoA:3-oxoacid-CoA transferase.

The “thrifty substrate” hypothesis was proposed in 2016 and suggested that application of SGLT inhibitors causes a reduction of body glucose and insulin/glucagon ratio, with subsequent lipid mobilisation and oxidation in the liver, stimulating ketogenesis (Figure 1). Such a metabolic state is like prolonged fasting and increases myocardial uptake of the primary ketone body-β-hydroxybutyrate. Metabolically, ATP production from ketone body oxidation is more efficient than FA oxidation and does not produce adverse side products [37,38]. Additionally, recent research suggested beneficial anti-pyroptotic [39] and antioxidant [40] effects for β-hydroxybutyrate, combined with a slight pro-inflammatory action on endothelial cells [41]. This hypothesis is well-supported by multiple research when SGLT2 application resulted in increased lipolysis and ketogenesis [42] and improvement of weight, blood pressure, and vascular parameters [43]. Similarly, many beneficial effects were described for heart failing non-diabetic rats: increased left ventricular ejection fraction, cardiomyocyte hypertrophy, diminished interstitial fibrosis, and reduced myocardial oxidative stress. Additionally, myocardial utilisation of ketone bodies was increased, uptake and oxidation of glucose and FAs were normalised, leading to increased cardiac ATP production [44].

However, several critical points are against the “thrifty substrate” hypothesis. One of the major concerns is the origin of generated high β-hydroxybutyrate levels, resulting from reduced oxidation in the heart and/or skeletal muscle [45].Recent research also suggested that high FA level upregulates GSK-3α (glycogen synthase kinase-3α), which phosphorylates PPARα and enhances transcription of a subset of PPARα targets, selectively stimulating FA uptake and storage, but not oxidation, thereby promoting lipid accumulation in cardiomyocytes, subsequently leading to lipotoxic cardiomyopathy [46].

Another concern is the beneficial effect of the metabolic switch toward ketone body oxidation. The old idea that the failing heart’s performance is reduced because of the lack of energy (“engine out of fuel”) and supply of more effective fuel (ketone bodies) would boost myocardial metabolism has been challenged many times [47]. However, several recent investigations support this idea. For example, the injection of 3-hydroxybutyrate had beneficial haemodynamic effects for both heart failure and reduced ejection fraction hemodynamic patients and healthy volunteers [48]. Similarly, data from the heart failure mice model system suggest the beneficial effect of chronic ketone ester supplementation (reduction of cardiac fibrosis and increased cardiac output) [49]. Another research study proposed that chronically elevated β-hydroxybutyrate level reduced cardiac NLRP3 inflammasome activation, thus modulating cardiac inflammation and protecting against heart failure development [50,51]. Chronic supplementation of the rodent with induced heart failure increased the expression of genes involved in ketone body utilisation and normalised myocardial ATP production, but with no effect on cardiac fibrosis [52]. Application of empagliflozin to non-diabetic male rats after induced cardiac arrest did not influence heart rate and blood pressure, however, left ventricular function and survival time were increased. In addition, the levels of myocardial fibrosis, serum cardiac troponin I levels, and myocardial oxidative stress were reduced, while the mitochondrial activity was increased. In general, cardiac energy metabolism was increased and associated with reduced glucose levels and increased ketone body oxidation metabolism. Thus, suggesting that empagliflozin could be beneficial for patients with myocardial dysfunction after cardiac arrest [53].

On the contrary, another study did not find any improvement in the cardiac efficiency after β-hydroxybutyrate perfusion. The beneficial effect was limited to increased overall energy production without compromising glucose or fatty acid metabolism [54]. However, exposure of rats to a prolonged ketogenic diet resulted in increased *SIRT7* (NAD-dependent protein-lysine deacylase 7) expression inhibited transcription of mitochondrial ribosome-encoding genes and mitochondrial biogenesis, which resulted in cardiomyocyte apoptosis and cardiac fibrosis [55]. Sirt7 is the crucial protein known to regulate mitochondrial function and biogenesis, coordinate glucose availability, and maintain energy homeostasis [56]. Further, experiments with empagliflozin-treated myocardial infarction induced DM mice suggested increased glucose oxidation and ketone utilisation with increased myocardial levels of Sirt3 (mitochondrial deacetylation modification enzyme, which promotes effective oxidative metabolism) and antioxidant enzyme SOD2 (Superoxide Dismutase 2) [57]. Similarly, the absence of competition between substrates (FAs, glucose, and ketone bodies) was observed in another study [58]. On the other hand, increased glucose level suppresses the cardiac ketolytic pathway through several mechanisms in the diabetic myocardium [59]. Thus, additional functional analysis is required to define the exact molecular mechanisms of the glucose-mediated effect on ketone body metabolism.

The therapeutic application of the ketone bodies is under intensive investigation, and future studies will clarify whether enhancing myocardial metabolic efficiency is beneficial for heart functions and what is the exact molecular mechanism of those effects [60,61].

### 2.3. The Sodium Hypothesis

SGLT2 inhibitors also affect tissue and cellular Na^+^ homeostasis. Na^+^ regulates Ca^2+^ cycling, mitochondrial redox regulation, and electrical activity in cardiac myocytes and plays a central role in excitation-contraction. Therefore, the equilibrium of myocyte Na^+^ homeostasis is perturbed in heart failure and diabetic hearts, resulting in higher [Na^+^]_i_ (Na^+^ intracellular) concentration. Elevated [Na^+^]_i_ causes oxidative stress and increases the sarcoplasmic reticulum Ca^2+^ leak, thus promoting heart dysfunction and increasing the risk for arrhythmias [62].

Failing cardiac myocytes have impairment in both Na^+^ and Ca^2+^: a decreased amplitude and velocity of [Ca^2+^]_c_ (cytosolic Ca^2+^) transients and increased diastolic [Ca^2+^]_c_ and [Na^+^]_i_. The predominant defects in Ca^2+^ and Na^+^ handling are caused by a reduced function of SERCA (sarco/endoplasmic reticulum Ca^2+^-ATPase), increased expression and activity of NCX (Na^+^/Ca^2+^-Exchange Protein 1), increased activity of the NHE (sarcolemmal Na^+^/H^+^ -exchanger), and reduced activity of NKA (Na^+^/K^+^-ATPase) [63].

A recent report suggested an SGLT2-independent effect of empagliflozin in reduction of [Na^+^]_c_ and [Ca^2+^]_c_ and enhancing [Ca^2+^]_m_ (mitochondrial Ca^2+^), which acts through impairment of myocardial NHE flux in isolated ventricular myocytes [64]. Treatment of diabetic rats with empagliflozin also affects Ca^2+^ regulation, late Na^+^ and Na^+^/H^+^-exchanger currents, normalises left ventricular end-diastolic diameters and QT intervals, and attenuates the prolonged action potential duration [65]. Application of empagliflozin in female db/db mice improved glycaemic indices, diastolic function and eccentric left ventricular hypertrophy. In addition, the expression of profibrotic/prohypertrophic genes (collagen I and III), *SGK1* (serum/glucocorticoid regulated kinase 1) and the *ENaC* (epithelial sodium channel) were reduced. However, without detectable changes in blood pressure [66,67]. Excess circulating glucose stimulates *SGK1* expression; thus, it is highly expressed in the diabetic heart. Further, SGK1 regulates many ion channels (including ENaC), transporters and enzymes, serving as a mediator of cardiac fibrosis and impaired cardiac relaxation [68]. Similarly, in angiotensin II-stressed diabetic mice, dapagliflozin attenuated fibrosis, and inflammation increased the left ventricular fractional shortening. In isolated cardiomyocytes, dapagliflozin decreased the expression of *NHE*, *NCX,* and *CACNA1C* (voltage-dependent L-type calcium channel or LTCC), thus connecting cardioprotection and modulation of Ca^2+^ ion homeostasis [69,70].

The molecular mechanism responsible for [Na^+^]_c_-lowering effect of SGLT2i (empagliflozin, dapagliflozin, canagliflozin), known to occur in heart failure and diabetes, is mediated through their direct binding with the Na^+^-binding site of NHE [71]. Application of sotagliflozin, an SGLT1 and 2 inhibitor [72], EU approved to treat T1DM [73], on a heart failure rat model system ameliorated left atrial enlargement, increased incidence and amplitude of arrhythmic SCaEs (spontaneous Ca^2+^ release events), reduced the magnitude of SCaEs, and increased NCX forward-mode activity. Sotagliflozino also enhanced mitochondrial Ca^2+^ buffer capacity, prevented mitochondrial swelling, and improved mitochondrial fission [74]. Additionally, dapagliflozin was shown to decrease the skin tissue sodium content [75], which is associated with left ventricular mass in patients with chronic kidney disease [76]. Furthermore, interesting results were obtained on metabolic syndrome rats, where treatment with dapagliflozin affects Zn^2+^ homeostasis by acting on Zn^2+^ transporters, cardiac matrix metalloproteinases, with decreased levels of oxidative stress [77]. Therefore, the proposed Zn^2+^-mediated cardioprotective effects of SGLT2 inhibitors could be a new area in exploring diabetic and failing heart model systems.

**Figure 2:**
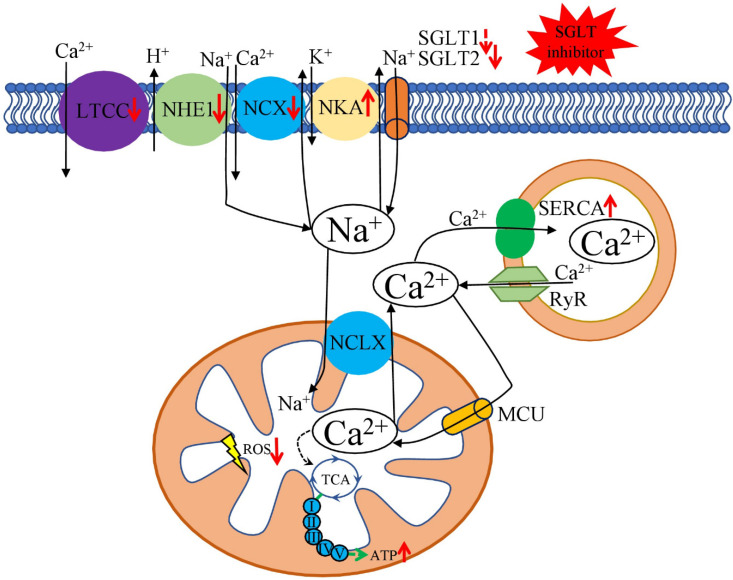
Beneficial effects of SGLT2 inhibitors on cardiomyocytes Na^+^ and Ca^2+^ metabolism. Through the different mechanisms, SGLT2 inhibitors reduce the activity of LTCC, NHE1, and NCX and increase the activity of NKA and SERCA. Such effects ameliorate DM-associated overload of [Na^+^]_c_ and [Ca^2+^]_c_ and enhance [Ca^2+^]_m_. [Na^+^]_c_ level is connected to the [Ca^2+^]_m_ through the mitochondrial Na^+^/Ca^2+^ exchanger (NCLX). However, the efficacy of NCLX is lower compared to the Ca^2+^ uptake by the MCU. [Ca^2+^]_c_ level is also affected by the Ca^2+^ uptake by SERCA and the leakage from the SR RyR receptors. [Ca^2+^]_m_ concentration regulates TCA cycle dehydrogenases, resulting in increased ATP and reduced ROS production. Red arrows show the effect of SGLT2 inhibitors on the key transporters; black arrows—flows of the discussed ions; green arrows—ATP production by the mitochondrial oxidative phosphorylation system. NHE—sarcolemmal Na^+^/H^+^-exchanger, LTCC—voltage-dependent L-type calcium channel, ATP—adenosine triphosphate, MCU—mitochondrial Ca^2+^ uniporter, NCX—sarcolemmal Na^+^/Ca^2+^ exchanger, NKA—Na^+^/K^+^-ATPase, RyR—ryanodine receptor, SERCA—sarcoplasmic reticulum Ca^2+^-ATPase.

Discussed results suggest that cardio beneficial effects of SGLT2 inhibitors, at least partially, could be explained by their direct impact on cardiac ion homeostasis—decreasing [Na^+^]_i_ and restoring [Ca^2+^]_m_ handling (Figure 2). Additionally, SGLT2 inhibitors influence ion homeostasis on the organism-wide level, suggesting that SGLT2 inhibitors could be helpful in treatments of other diseases in which development and pathophysiology involve ion dysregulation.

We could conclude that SGLT2 inhibitors probably act in a pleiotropic way on the heart, kidney, and skin. As a possible scenario, the SGLT2 inhibitor-mediated cardio beneficial effect as a combination of all three discussed hypotheses, altogether provides overall positive outcomes. It is important to note that many studies were conducted in vitro or on animal model systems, which have certain limitations and not always could the obtained knowledge be extrapolated on an organism-wide level.

## 3. Effect of SGLT2 Inhibitors on Mitochondria

Although the cardiovascular benefits of SGLT2 inhibition were confirmed by multiple research studies, the underlying molecular mechanisms are still debated. Besides the mechanisms discussed in the previous section, several exciting directions are being explored for SGLT inhibitor-mediated modulation of mitochondria function and metabolism in different organs and tissues (primarily heart and kidney). It is known that mitochondrial dysfunction plays a central role in both diabetic cardiomyopathies and heart failure, with several therapeutic strategies having been developed to target specifically cardiac mitochondria [78,79].

The primary function of mitochondria is to provide cells with energy in the form of ATP. Another crucial function is the regulation of Ca^2+^ homeostasis and Ca^2+^ regulated processes (such as signalling, proliferation, cell cycle, and respiratory bioenergetics) [80,81]. Mitochondria are also involved in regulating immune signalling and apoptosis, hormonal signalling, and steroid biosynthesis [82,83,84,85,86].

The effectiveness of mitochondria is controlled via several processes, such as biogenesis, turnover, and recycling. Mitochondrial biogenesis could be activated by environmental stimuli, several types of cellular stress, and developmental signals, with PGC-1α (peroxisome proliferator-activated receptor gamma coactivator 1-alpha) as the central regulator of mitochondrial biogenesis [87]. Mitochondria are equally distributed between daughter cells during cellular division, while damaged and dysfunctional mitochondria are salvaged with a specialised form of autophagy–mitophagy. Damaged and dysfunctional parts of mitochondria are separated for mitophagy during mitochondrial turnover, which comprises cycles of fission (split) and fusion (merge). Mitochondrial fission is regulated by *DNM1L* (dynamin 1 like) and *FIS1* (fission, mitochondrial 1), genes and *MFN1*, *MFN2* (mitofusin 1 and 2), and *OPA1* (optic atrophy protein 1) are responsible for fusion. Further, lysosomes fuse with separated dysfunctional mitochondria and digest them, while the healthy parts of mitochondria are fused back to the mitochondrial network and continue normal functioning [88]. Mitophagy is crucial for cell differentiation and embryonic development, apoptosis, inflammation, and numerous other processes; its impairment is associated with many neurodegenerative diseases, pathological ageing, and inflammageing, cancer and other conditions [89,90].

The following section summarises recent evidence of SGLT2 inhibitors’ positive effects on mitochondrial function, dynamics, and metabolism (Table 1).

Dysfunctional mitochondria produce more harmful ROS and generate less ATP. Surplus mROS and decreased ATP production could dysregulate cardiac functions in several ways:

1. mROS modulates redox-sensitive regulatory domains of several proteins involved in excitation-contraction coupling (such as NCX, SERCA, LCCs (L-type Ca^2+^-channels), Na^+^-channels, K^+^-channels, RyRs (ryanodine receptors), and others—reviewed in [105,106,107].
2. mROS could directly activate CaMKII (Ca^2+^/calmodulin dependent kinase II), a multifunctional nodal regulator of many cellular pathways, including excitation-contraction coupling [108].
3. increased mROS combined with hyperglycaemia provide persistent CaMKII activation, a major driver of arrhythmogenicity in diabetic hearts [109].
4. chronic hyperglycaemia and CaMKII activation downregulate K^+^ channel expression and function in the NOX2-ROS-PKC (NADPH oxidase 2–ROS-protein kinase C) pathway, which increases arrhythmia risk [110].
5. low ATP levels could suppress the activity of SERCA and Na^+^/K^+^-ATPase, which will alter Ca^2+^ homeostasis and increase arrhythmia risk [111].
6. such pathological deficiencies could promote cardiomyocyte hypertrophy and interstitial fibrosis, two critical drivers of arrhythmia—reviewed in [112].

**Figure 3:**
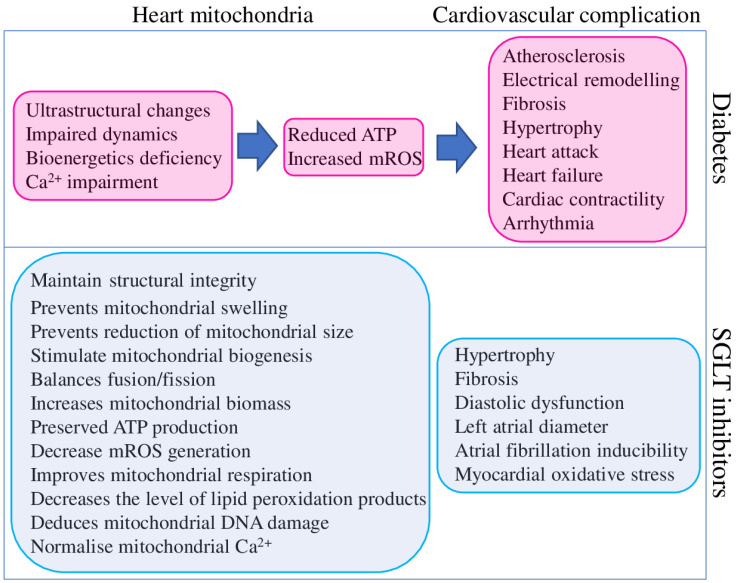
Contribution of diabetes to cardiovascular complications mediated through heart mitochondria dysfunction (magenta) and beneficial effects of SGLT2 inhibitors on mitochondria function and cardiovascular system (blue).

Mitochondrial dysfunction has been described in the heart and other organs of patients with T2DM, metabolic syndrome, obesity, and related animal model systems and cell cultures. Identified dysfunctions are represented by reduced mitochondrial respiration, increased mROS production and mtDNA damage, abnormal mitochondrial structure, altered dynamics and metabolism, and bioenergetic and biogenesis deficiencies (Figure 3).

In total, diabetes-associated mitochondrial dysfunction could cause cardiovascular complications by several mechanisms, as summarised in Figure 3. At the same time, the same findings suggest that targeting mitochondria could represent a valuable therapeutic strategy to reduce the burden of cardiovascular complications in diabetic patients [106]. Although SGLT2 inhibitors were explicitly designed to reduce hyperglycaemia in T2DM patients, they clearly improve the mitochondrial function of different organs and tissues of non-diabetic animal models. SGLT2 inhibitors provide a positive effect on mitochondrial biogenesis (through the up-regulation of the critical transcription factors PGC-1α and TFAM), fission/fusion balance and mitophagy (through the regulation of DNM1L, FIS1, MFN1, MFN2, and OPA1), ion homeostasis (through the regulation of SGK1 and direct effect on ion channels) metabolism (substrate preferences and utilisation, ATP and mROS output), and structural integrity. Such broad and diverse beneficial effects of SGLT2 inhibitors on mitochondria suggest their high potential for treating non-diabetic diseases.

## 4. Conclusions

SGLT2 inhibitors are a new and intensively studied class of medications used for T2DM treatment. Recent research and clinical evidence showed that SGLT2 inhibitors could reduce the incidence of cardiovascular complications in both T2DM and non-DM patients with high efficacy, thus suggesting the potential role of SGLT2 inhibitors as a treatment of CVD. Therefore, we can conclude that the beneficial effects of SGLT2 inhibitors on the cardiovascular system are mediated by improving mitochondrial function and restoring ion homeostasis. Several promising SGLT2 inhibitor-based T2DM therapies with CVD-protecting activities are currently in development. However, given the highly diverse nature of metabolic pathways involved in T2DM pathogenesis and the development of associated cardiovascular complications, a clear understanding of the underlying molecular mechanisms is required to provide adequate care and treatment. Despite significant success in understanding SGLT2 inhibitors’ pleiotropic activities, further collaboration between clinical and basic science researchers is necessary to determine the exact molecular mechanism of their action on the cardiovascular system. Such research would allow a more comprehensive application of SGLT2 inhibitors also for CVD treatment.

## Figures and Tables

**Table 1:** Effect of SGLT2 inhibitors on mitochondrial function and associated cardiovascular benefits.

Used Drug	Experimental System/Model Animal/Cell Culture	Cardiovascular Effect	Mitochondrial Effects	Other Effects/Notes	References
Empagliflozin	non-DM male rats after CA	increases LV function and survival time; reduces myocardial fibrosis, serum cardiac troponin I levels and myocardial OS after CA	maintains the structural integrity of myocardial mitochondria and increases mitochondrial activity after CA	increases circulating and myocardial ketone levels and heart *BDH1* expression	[53]
Empagliflozin	DM rats after MI	the sizes of MI were comparable	increases myocardial levels of Sirt3	increases glucose oxidation and ketone utilisation, SOD2 levels	[57]
Empagliflozin	in vitro culture of ventricular myocytes (rabbits and rats)	-	enhances [Ca^2+^]_m_	reduces [Na^+^]_c_ and [Ca^2+^]_c_	[64]
Sotagliflozin	obese rats’ model of HFpEF	ameliorates LA enlargement in HFpEF in vivo; reduced the magnitude of SCaEs in-vitro LA cardiomyocytes	prevents mitochondrial swelling, enhances mitochondrial Ca^2+^ buffer capacity, improves mitochondrial fission and ROS production, averts Ca^2+^ accumulation upon glycolytic inhibition; increases NCX forward-mode activity	lowers diastolic [Ca^2+^] of CaT	[74]
Ertugliflozin	mice on HFD and HSD	beneficial for hallmarks of DCMP: LV hypertrophy, myocyte hypertrophy, myocardial interstitial fibrosis, and diastolic dysfunction	prevents mitochondrial dysfunction, preserves ATP production, and decreases mROS generation	positive enrichment of gene sets related to OXPHOS (oxidative phosphorylation) and FAM	[91]
Empagliflozin	streptozotocin-induced DM mice	improves diabetic myocardial structure and function, preserves cardiac microvascular barrier function and integrity, sustains eNOS phosphorylation and endothelium-dependent relaxation, improves microvessel density and perfusion	inhibits mitochondrial fission, suppresses mROS production	preserves CMEC barrier function and impedes CMEC senescence	[92]
Empagliflozin	myocardial tissues of the DM rats after MI	-	suppresses *FIS1* and increases *BNIP3* expression; prevents reduction of mitochondrial size and autophagic vacuole number; upregulates *SOD2* and *CAT* expression	reduces blood glucose and triglycerides, increases lipid droplets in cardiomyocytes	[93]
Dapagliflozin	overweight insulin-resistant MetS-rats	augments the increased blood pressure, prolonged Q–R interval, and low heart rate with depressed LV function and relaxation of the aorta	preserves the depolarised mitochondrial membrane potential; normalises the expression of fusion-fission proteins and cytosolic Ca^2+^-homeostasis	increases voltage-gated Na^+^-currents and intracellular pH; normalises the cellular levels of increased OS, protein–thiol oxidation and ADP/ATP ratio in cardiomyocytes	[94]
Empagliflozin	streptozotocin- induced HFD DM rats	reduces left atrial diameter, interstitial fibrosis, and the incidence of AF inducibility	improves atrial mitochondrial respiratory function, mitochondrial membrane potential, and mitochondrial biogenesis	increases the expression of *PGC-1a*, *NRF-1* and *TFAM* (Transcription Factor A, Mitochondrial)	[95]
Empagliflozin	streptozotocin-induced DM mice; hRPTCs	-	improves mitochondrial biogenesis and balances fusion–fission proteins expression; increases autophagy; reduces mROS and expression of apoptotic and fibrotic proteins in hRPTCs; normalises AMP/ATP ratios	suppresses *SGLT2* expression and ameliorates renal morphological changes in the kidneys of DM mice	[96]
Empagliflozin	mice with HFD-induced lipid overload	-	normalises mitochondrial function in the heart via an increase in FAO and protects against HFD-induced disturbances in cardiac metabolism	increases palmitate uptake and decreases the accumulation of metabolites of incomplete FAO in cardiac tissues	[97]
Ipragliflozin	HFD mice	-	normalises mitochondrial morphology and fusion restores *OPA1* and *MFN2* expression; reduces mROS	ameliorates tubular vacuolation, dilatation and epithelial cell detachment	[98]
Empagliflozin	DM mice	-	alleviates mitochondrial fission via AMPK/SP1/PGAM5 pathway	renal protection in DKD	[99]
Dapagliflozin	HFD-induced obese rats	-	improves brain mitochondria function, insulin signalling, apoptosis and prevents cognitive decline	improves peripheral insulin sensitivity and hippocampal synaptic plasticity, reduces weight gain	[100]
Dapagliflozin	hepatocytes of HFD streptozotocin-induced DM mice	-	prevents mitochondrial swelling; normalises the mitochondrial size, mtDNA copy number and mitochondrial respiration; decreases the level of lipid peroxidation products in mitochondria	increases the expression of the *MFN2* and *DRP1* in the liver tissue;	[101]
Empagliflozin	human cardiomyocyte cells; CAG-RFP-EGFP-LC3, Becn1^+/−^, SIRT3-knock-out and TLR9-knock-out mice	protects against doxorubicin-induced cardiomyopathy through a mitochondrial TLR9-SIRT3 mechanism; increases autophagic flux in hearts and cardiomyocytes	increases the TLR9 activation and the abundance of SIRT3 in the mitochondria, which enhances the mitochondrial respiration rate and exerts its protection against ROS and apoptosis	-	[102]
Empagliflozin	non-DM rats with LV dysfunction after MI	increases the LV ejection fraction, attenuates cardiomyocyte hypertrophy, diminishes interstitial fibrosis and reduces myocardial OS	reduces mitochondrial DNA damage and stimulated mitochondrial biogenesis, normalises the myocardial uptake and oxidation of glucose and fatty acids	increases urine production two-fold without affecting creatinine clearance and serum electrolytes; increases circulating ketone levels and myocardial expression of the *MCT1* and *BDH1*	[44]
Empagliflozin	non-DM rats after MI	increases cardiac contractility and improves systolic heart function after MI; does not affect arterial stiffness, blood pressure, markers of fibrosis, and necroptosis;	NHE1 modulation decreases [Na^+^]_c_ and [Ca^2+^]_c_ levels while increasing the myocytes [Ca^2+^]_m_ concentra-tion	inhibits *MMP9*, down-regulates *NHE1* and upregulates *SERCA2a* expression	[103]
Empagliflozin	wild-type and Parkin^−/−^ male mice after PCAL; H9C2 cells	attenuates PCAL-induced adverse remodelling	increases mitochondrial biomass, respiratory capacity, and markers of mitochondrial biogenesis; the mechanism is not entirely de-pendent on Parkin	-	[104]

## Data Availability

Not applicable.

## Abbreviations

AcAc CoA	acetoacetyl CoA
ACAT1	acetyl-CoA acetyltransferase
AF	atrial fibrillation
ATP	adenosine triphosphate
BDH1	mitochondrial β-hydroxybutyrate dehydrogenase
C2-carnitine	acetylcarnitine,
C4-OH carnitine	hydroxy butyryl carnitine
CA	cardiac arrest
CACNA1C	voltage-dependent L-type calcium channel or LTCC
CaMKII	Ca^2+^/calmodulin dependent kinase II
CaT	Ca^2+^ transients
CMEC	cardiac microvascular endothelial cell
CPT1	carnitine palmitoyltransferase 1
CVD	cardiovascular diseases
DCMP	diabetic cardiomyopathy
DKD	diabetic kidney disease
DNM1L	dynamin 1 like
FA	fatty acids
FAM	fatty acid metabolism
FAO	fatty acid oxidation
FIS1	fission, mitochondrial 1
GSK-3α	glycogen synthase kinase-3α
HFD	high-fat diet
HfpEF	heart failure with a preserved ejection fraction
HMGCL	3-hydroxy-3-methylglutaryl-coenzyme A lyase
HMG-CoA	3-hydroxy-3-methtylglutaryl-CoA
hRPTCs	human renal proximal tubular cells
HSD	high-sucrose diet
LA	left atrial
LCCs	L-type Ca^2+^-channels
MCT1	monocarboxylate transporter 1
MCU	mitochondrial Ca^2+^ uniporter
MFN1, MFN2	mitofusin 1 and 2
NCLX	mitochondrial Na+/Ca^2+^ exchanger
MMP9	matrix metalloproteinase 9
mROS	mitochondrial reactive oxygen species
NCX1	sarcolemmal Na^+^/Ca^2+^-Exchange Protein 1
NHE	sarcolemmal Na^+^/H^+^ -exchanger
NKA	Na^+^/K^+^-ATPase
NOX2	NADPH oxidase 2
OPA1	optic atrophy protein 1
OS	oxidative stress
OXPHOS	oxidative phosphorylation
PCAL	permanent coronary artery ligation
PGC-1α	peroxisome proliferator-activated receptor gamma coactivator 1-alpha
PKC	protein kinase C
PPAR-α	peroxisome proliferator-activated receptor alpha
RyR	ryanodine receptor
ScaEs	spontaneous Ca^2+^ release events
SCaEs	spontaneous Ca^2+^ release events
SCOT	Succinyl-CoA:3-oxoacid-CoA transferase
SIRT7	NAD-dependent protein-lysine deacylase 7
SERCA	sarco/endoplasmic reticulum Ca^2+^-ATPase
SGK1	serum/glucocorticoid regulated kinase 1
SGLTSOD2	Na^+^-glucose co-transportersuperoxide dismutase 2
TCA	tricarboxylic acid cycle
T2DMTFAM	type 2 diabetes mellitus transcription factor A, mitochondrial
βOHB	β-hydroxybutyrate

## References

[1] International Diabetes Federation (2021). IDF Diabetes Atlas.

[2] American Diabetes Association (2019). Cardiovascular Disease and Risk Management: Standards of Medical Care in Diabetes-2019. Diabetes Care.

[3] Summerhill V.I., Grechko A.V., Yet S.F., Sobenin I.A., Orekhov A.N. (2019). The Atherogenic Role of Circulating Modified Lipids in Atherosclerosis. Int. J. Mol. Sci..

[4] Laiteerapong N., Ham S.A., Gao Y., Moffet H.H., Liu J.Y., Huang E.S., Karter A.J. (2019). The Legacy Effect in Type 2 Diabetes: Impact of Early Glycemic Control on Future Complications (The Diabetes & Aging Study). Diabetes Care.

[5] Tian J., Ohkuma T., Cooper M., Harrap S., Mancia G., Poulter N., Wang J.-G., Zoungas S., Woodward M., Chalmers J. (2020). Effects of Intensive Glycemic Control on Clinical Outcomes Among Patients With Type 2 Diabetes With Different Levels of Cardiovascular Risk and Hemoglobin A1c in the ADVANCE Trial. Diabetes Care.

[6] Ohkuma T., Chalmers J., Cooper M., Hamet P., Harrap S., Marre M., Mancia G., Poulter N., Woodward M. (2021). The Comparative Effects of Intensive Glucose Lowering in Diabetes Patients Aged below or above 65 Years: Results from the ADVANCE Trial. Diabetes Obes. Metab..

[7] Reaven P.D., Emanuele N.V., Wiitala W.L., Bahn G.D., Reda D.J., McCarren M., Duckworth W.C., Hayward R.A. (2019). VADT Investigators Intensive Glucose Control in Patients with Type 2 Diabetes—15-Year Follow-Up. N. Engl. J. Med..

[8] Crane R.K. (1960). Intestinal Absorption of Sugars. Physiol. Rev..

[9] Hediger M.A., Coady M.J., Ikeda T.S., Wright E.M. (1987). Expression Cloning and CDNA Sequencing of the Na+/Glucose Co-Transporter. Nature.

[10] Ghezzi C., Loo D.D.F., Wright E.M. (2018). Physiology of Renal Glucose Handling via SGLT1, SGLT2 and GLUT2. Diabetologia.

[11] Sędzikowska A., Szablewski L. (2021). Human Glucose Transporters in Renal Glucose Homeostasis. Int. J. Mol. Sci..

[12] FDA (2018). Sodium-Glucose Cotransporter-2 (SGLT2) Inhibitors.

[13] Vallon V., Thomson S.C. (2017). Targeting Renal Glucose Reabsorption to Treat Hyperglycaemia: The Pleiotropic Effects of SGLT2 Inhibition. Diabetologia.

[14] Perkovic V., Jardine M.J., Neal B., Bompoint S., Heerspink H.J.L., Charytan D.M., Edwards R., Agarwal R., Bakris G., Bull S. (2019). Canagliflozin and Renal Outcomes in Type 2 Diabetes and Nephropathy. N. Engl. J. Med..

[15] Wanner C., Inzucchi S.E., Lachin J.M., Fitchett D., von Eynatten M., Mattheus M., Johansen O.E., Woerle H.J., Broedl U.C., Zinman B. (2016). Empagliflozin and Progression of Kidney Disease in Type 2 Diabetes. N. Engl. J. Med..

[16] Heerspink H.J.L., Stefánsson B.V., Correa-Rotter R., Chertow G.M., Greene T., Hou F.-F., Mann J.F.E., McMurray J.J.V., Lindberg M., Rossing P. (2020). Dapagliflozin in Patients with Chronic Kidney Disease. N. Engl. J. Med..

[17] Pan D., Xu L., Chen P., Jiang H., Shi D., Guo M. (2021). Empagliflozin in Patients With Heart Failure: A Systematic Review and Meta-Analysis of Randomized Controlled Trials. Front. Cardiovasc. Med..

[18] Ehrenkranz J.R.L., Lewis N.G., Kahn C.R., Roth J. (2005). Phlorizin: A Review. Diabetes Metab. Res. Rev..

[19] Grempler R., Thomas L., Eckhardt M., Himmelsbach F., Sauer A., Sharp D.E., Bakker R.A., Mark M., Klein T., Eickelmann P. (2012). Empagliflozin, a Novel Selective Sodium Glucose Cotransporter-2 (SGLT-2) Inhibitor: Characterisation and Comparison with Other SGLT-2 Inhibitors. Diabetes Obes. Metab..

[20] Kwak S.H., Hwang Y.-C., Won J.C., Bae J.C., Kim H.J., Suh S., Lee E.Y., Lee S., Kim S.-Y., Kim J.H. (2020). Comparison of the Effects of Gemigliptin and Dapagliflozin on Glycaemic Variability in Type 2 Diabetes: A Randomized, Open-Label, Active-Controlled, 12-Week Study (STABLE II Study). Diabetes Obes. Metab..

[21] McNeill A.M., Davies G., Kruger E., Kowal S., Reason T., Ejzykowicz F., Hannachi H., Cater N., McLeod E. (2019). Ertugliflozin Compared to Other Anti-Hyperglycemic Agents as Monotherapy and Add-on Therapy in Type 2 Diabetes: A Systematic Literature Review and Network Meta-Analysis. Diabetes Ther..

[22] Kanbay M., Tapoi L., Ureche C., Tanriover C., Cevik E., Demiray A., Afsar B., Cherney D.Z.I., Covic A. (2021). Effect of Sodium-Glucose Cotransporter 2 Inhibitors on Hemoglobin and Hematocrit Levels in Type 2 Diabetes: A Systematic Review and Meta-Analysis. Int. Urol. Nephrol..

[23] Ott C., Jumar A., Striepe K., Friedrich S., Karg M.V., Bramlage P., Schmieder R.E. (2017). A Randomised Study of the Impact of the SGLT2 Inhibitor Dapagliflozin on Microvascular and Macrovascular Circulation. Cardiovasc. Diabetol..

[24] Van Bommel E.J.M., Muskiet M.H.A., van Baar M.J.B., Tonneijck L., Smits M.M., Emanuel A.L., Bozovic A., Danser A.H.J., Geurts F., Hoorn E.J. (2020). The Renal Hemodynamic Effects of the SGLT2 Inhibitor Dapagliflozin Are Caused by Post-Glomerular Vasodilatation Rather than Pre-Glomerular Vasoconstriction in Metformin-Treated Patients with Type 2 Diabetes in the Randomized, Double-Blind RED Trial. Kidney Int..

[25] Tang H., Zhang X., Zhang J., Li Y., Del Gobbo L.C., Zhai S., Song Y. (2016). Elevated Serum Magnesium Associated with SGLT2 Inhibitor Use in Type 2 Diabetes Patients: A Meta-Analysis of Randomised Controlled Trials. Diabetologia.

[26] Birkenfeld A.L., Jordan J., Dworak M., Merkel T., Burnstock G. (2019). Myocardial Metabolism in Heart Failure: Purinergic Signalling and Other Metabolic Concepts. Pharmacol. Ther..

[27] Song J.D., Alves T.C., Befroy D.E., Perry R.J., Mason G.F., Zhang X.-M., Munk A., Zhang Y., Zhang D., Cline G.W. (2020). Dissociation of Muscle Insulin Resistance from Alterations in Mitochondrial Substrate Preference. Cell Metabolism.

[28] Sobenin I.A., Salonen J.T., Zhelankin A.V., Melnichenko A.A., Kaikkonen J., Bobryshev Y.V., Orekhov A.N. (2014). Low Density Lipoprotein-Containing Circulating Immune Complexes: Role in Atherosclerosis and Diagnostic Value. BioMed Res. Int..

[29] Mereweather L.J., Montes Aparicio C.N., Heather L.C. (2020). Positioning Metabolism as a Central Player in the Diabetic Heart. J. Lipid Atheroscler..

[30] Strand E., Lysne V., Grinna M.L., Bohov P., Svardal A., Nygård O., Berge R.K., Bjørndal B. (2019). Short-Term Activation of Peroxisome Proliferator-Activated Receptors α and γ Induces Tissue-Specific Effects on Lipid Metabolism and Fatty Acid Composition in Male Wistar Rats. PPAR Res..

[31] Sikder K., Shukla S.K., Patel N., Singh H., Rafiq K. (2018). High Fat Diet Upregulates Fatty Acid Oxidation and Ketogenesis via Intervention of PPAR-γ. Cell. Physiol. Biochem..

[32] Toral M., Romero M., Pérez-Vizcaíno F., Duarte J., Jiménez R. (2017). Antihypertensive Effects of Peroxisome Proliferator-Activated Receptor-β/δ Activation. Am. J. Physiol. Heart Circ. Physiol..

[33] Papatheodorou I., Galatou E., Panagiotidis G.-D., Ravingerová T., Lazou A. (2021). Cardioprotective Effects of PPARβ/δ Activation against Ischemia/Reperfusion Injury in Rat Heart Are Associated with ALDH2 Upregulation, Amelioration of Oxidative Stress and Preservation of Mitochondrial Energy Production. Int. J. Mol. Sci..

[34] Shao M., Wang M., Ma L., Wang Q., Gao P., Tian X., Li C., Lu L., Li C., Wang W. (2021). β-Elemene Blocks Lipid-Induced Inflammatory Pathways via PPARβ Activation in Heart Failure. Eur. J. Pharmacol..

[35] Karwi Q.G., Sun Q., Lopaschuk G.D. (2021). The Contribution of Cardiac Fatty Acid Oxidation to Diabetic Cardiomyopathy Severity. Cells.

[36] Chistiakov D., Revin V., Sobenin I., Orekhov A., Bobryshev Y. (2015). Vascular Endothelium: Functioning in Norm, Changes in Atherosclerosis and Current Dietary Approaches to Improve Endothelial Function. MRMC.

[37] Ferrannini E., Mark M., Mayoux E. (2016). CV Protection in the EMPA-REG OUTCOME Trial: A “Thrifty Substrate” Hypothesis. Diabetes Care.

[38] Mudaliar S., Alloju S., Henry R.R. (2016). Can a Shift in Fuel Energetics Explain the Beneficial Cardiorenal Outcomes in the EMPA-REG OUTCOME Study? A Unifying Hypothesis. Diabetes Care.

[39] Tajima T., Yoshifuji A., Matsui A., Itoh T., Uchiyama K., Kanda T., Tokuyama H., Wakino S., Itoh H. (2019). β-Hydroxybutyrate Attenuates Renal Ischemia-Reperfusion Injury through Its Anti-Pyroptotic Effects. Kidney Int..

[40] Qiu X., Rong X., Yang J., Lu Y. (2019). Evaluation of the Antioxidant Effects of Different Histone Deacetylase Inhibitors (HDACis) on Human Lens Epithelial Cells (HLECs) after UVB Exposure. BMC Ophthalmol..

[41] Chriett S., Dąbek A., Wojtala M., Vidal H., Balcerczyk A., Pirola L. (2019). Prominent Action of Butyrate over β-Hydroxybutyrate as Histone Deacetylase Inhibitor, Transcriptional Modulator and Anti-Inflammatory Molecule. Sci. Rep..

[42] Szekeres Z., Toth K., Szabados E. (2021). The Effects of SGLT2 Inhibitors on Lipid Metabolism. Metabolites.

[43] Pietschner R., Kolwelter J., Bosch A., Striepe K., Jung S., Kannenkeril D., Ott C., Schiffer M., Achenbach S., Schmieder R.E. (2021). Effect of Empagliflozin on Ketone Bodies in Patients with Stable Chronic Heart Failure. Cardiovasc. Diabetol..

[44] Yurista S.R., Silljé H.H.W., Oberdorf-Maass S.U., Schouten E.-M., Pavez Giani M.G., Hillebrands J.-L., van Goor H., van Veldhuisen D.J., de Boer R.A., Westenbrink B.D. (2019). Sodium-Glucose Co-Transporter 2 Inhibition with Empagliflozin Improves Cardiac Function in Non-Diabetic Rats with Left Ventricular Dysfunction after Myocardial Infarction. Eur. J. Heart Fail..

[45] Lopaschuk G.D., Verma S. (2016). Empagliflozin’s Fuel Hypothesis: Not so Soon. Cell Metab..

[46] Nakamura M., Liu T., Husain S., Zhai P., Warren J.S., Hsu C.-P., Matsuda T., Phiel C.J., Cox J.E., Tian B. (2019). Glycogen Synthase Kinase-3α Promotes Fatty Acid Uptake and Lipotoxic Cardiomyopathy. Cell Metab..

[47] Neubauer S. (2007). The Failing Heart—An Engine out of Fuel. N. Engl. J. Med..

[48] Nielsen R., Møller N., Gormsen L.C., Tolbod L.P., Hansson N.H., Sorensen J., Harms H.J., Frøkiær J., Eiskjaer H., Jespersen N.R. (2019). Cardiovascular Effects of Treatment With the Ketone Body 3-Hydroxybutyrate in Chronic Heart Failure Patients. Circulation.

[49] Takahara S., Soni S., Phaterpekar K., Kim T.T., Maayah Z.H., Levasseur J.L., Silver H.L., Freed D.H., Ferdaoussi M., Dyck J.R.B. (2021). Chronic Exogenous Ketone Supplementation Blunts the Decline of Cardiac Function in the Failing Heart. ESC Heart Fail..

[50] Byrne N.J., Soni S., Takahara S., Ferdaoussi M., Al Batran R., Darwesh A.M., Levasseur J.L., Beker D., Vos D.Y., Schmidt M.A. (2020). Chronically Elevating Circulating Ketones Can Reduce Cardiac Inflammation and Blunt the Development of Heart Failure. Circ. Heart Fail..

[51] Chistiakov D.A., Sobenin I.A., Orekhov A.N., Bobryshev Y.V. (2015). Myeloid Dendritic Cells: Development, Functions, and Role in Atherosclerotic Inflammation. Immunobiology.

[52] Yurista S.R., Matsuura T.R., Silljé H.H.W., Nijholt K.T., McDaid K.S., Shewale S.V., Leone T.C., Newman J.C., Verdin E., van Veldhuisen D.J. (2021). Ketone Ester Treatment Improves Cardiac Function and Reduces Pathologic Remodeling in Preclinical Models of Heart Failure. Circ. Heart Fail..

[53] Tan Y., Yu K., Liang L., Liu Y., Song F., Ge Q., Fang X., Yu T., Huang Z., Jiang L. (2021). Sodium–Glucose Co-Transporter 2 Inhibition With Empagliflozin Improves Cardiac Function After Cardiac Arrest in Rats by Enhancing Mitochondrial Energy Metabolism. Front. Pharmacol..

[54] Ho K.L., Zhang L., Wagg C., Al Batran R., Gopal K., Levasseur J., Leone T., Dyck J.R.B., Ussher J.R., Muoio D.M. (2019). Increased Ketone Body Oxidation Provides Additional Energy for the Failing Heart without Improving Cardiac Efficiency. Cardiovasc. Res..

[55] Xu S., Tao H., Cao W., Cao L., Lin Y., Zhao S.-M., Xu W., Cao J., Zhao J.-Y. (2021). Ketogenic Diets Inhibit Mitochondrial Biogenesis and Induce Cardiac Fibrosis. Signal. Transduct. Target. Ther..

[56] Yan W.-W., Liang Y.-L., Zhang Q.-X., Wang D., Lei M.-Z., Qu J., He X.-H., Lei Q.-Y., Wang Y.-P. (2018). Arginine Methylation of SIRT7 Couples Glucose Sensing with Mitochondria Biogenesis. EMBO Rep..

[57] Oshima H., Miki T., Kuno A., Mizuno M., Sato T., Tanno M., Yano T., Nakata K., Kimura Y., Abe K. (2019). Empagliflozin, an SGLT2 Inhibitor, Reduced the Mortality Rate after Acute Myocardial Infarction with Modification of Cardiac Metabolomes and Antioxidants in Diabetic Rats. J. Pharmacol. Exp. Ther..

[58] Monzo L., Sedlacek K., Hromanikova K., Tomanova L., Borlaug B.A., Jabor A., Kautzner J., Melenovsky V. (2021). Myocardial Ketone Body Utilization in Patients with Heart Failure: The Impact of Oral Ketone Ester. Metabolism.

[59] Brahma M.K., Ha C.-M., Pepin M.E., Mia S., Sun Z., Chatham J.C., Habegger K.M., Abel E.D., Paterson A.J., Young M.E. (2020). Increased Glucose Availability Attenuates Myocardial Ketone Body Utilization. J. Am. Heart Assoc..

[60] Yurista S.R., Nguyen C.T., Rosenzweig A., de Boer R.A., Westenbrink B.D. (2021). Ketone Bodies for the Failing Heart: Fuels That Can Fix the Engine?. Trends Endocrinol. Metab..

[61] Takahara S., Soni S., Maayah Z.H., Ferdaoussi M., Dyck J.R.B. (2021). Ketone Therapy for Heart Failure: Current Evidence for Clinical Use. Cardiovasc. Res..

[62] Despa S. (2018). Myocyte [Na+]i Dysregulation in Heart Failure and Diabetic Cardiomyopathy. Front. Physiol..

[63] Trum M., Riechel J., Wagner S. (2021). Cardioprotection by SGLT2 Inhibitors-Does It All Come Down to Na+?. Int. J. Mol. Sci..

[64] Baartscheer A., Schumacher C.A., Wüst R.C.I., Fiolet J.W.T., Stienen G.J.M., Coronel R., Zuurbier C.J. (2017). Empagliflozin Decreases Myocardial Cytoplasmic Na+ through Inhibition of the Cardiac Na+/H+ Exchanger in Rats and Rabbits. Diabetologia.

[65] Lee T.-I., Chen Y.-C., Lin Y.-K., Chung C.-C., Lu Y.-Y., Kao Y.-H., Chen Y.-J. (2019). Empagliflozin Attenuates Myocardial Sodium and Calcium Dysregulation and Reverses Cardiac Remodeling in Streptozotocin-Induced Diabetic Rats. Int. J. Mol. Sci..

[66] Habibi J., Aroor A.R., Sowers J.R., Jia G., Hayden M.R., Garro M., Barron B., Mayoux E., Rector R.S., Whaley-Connell A. (2017). Sodium Glucose Transporter 2 (SGLT2) Inhibition with Empagliflozin Improves Cardiac Diastolic Function in a Female Rodent Model of Diabetes. Cardiovasc. Diabetol..

[67] Soldatov V.O., Malorodova T.N., Balamutova T.I., Ksenofontov A.O., Dovgan A.P., Urozhevskaya Z.S. (2018). Endothelial Dysfunction: Comparative Evaluation of Ultrasound Dopplerography, Laser Dopplerflowmetry and Direct Monitoring of Arterial Pressure for Conducting Pharmacological Tests in Rats. RRP.

[68] Sierra-Ramos C., Velazquez-Garcia S., Vastola-Mascolo A., Hernández G., Faresse N., Alvarez de la Rosa D. (2020). SGK1 Activation Exacerbates Diet-Induced Obesity, Metabolic Syndrome and Hypertension. J. Endocrinol..

[69] Arow M., Waldman M., Yadin D., Nudelman V., Shainberg A., Abraham N.G., Freimark D., Kornowski R., Aravot D., Hochhauser E. (2020). Sodium-Glucose Cotransporter 2 Inhibitor Dapagliflozin Attenuates Diabetic Cardiomyopathy. Cardiovasc. Diabetol..

[70] Puchenkova O.A., Nadezhdin S.V., Soldatov V.O., Zhuchenko M.A., Korshunova D.S., Kubekina M.V., Korshunov E.N., Korokina L.V., Golubinskaya P.A., Kulikov A.L. (2020). STUDY OF Antiatherosclerotic And Endothelioprotective Activity of Peptide Agonists of Epor/Cd131 Heteroreceptor. Farm. Farmakol..

[71] Uthman L., Baartscheer A., Bleijlevens B., Schumacher C.A., Fiolet J.W.T., Koeman A., Jancev M., Hollmann M.W., Weber N.C., Coronel R. (2018). Class Effects of SGLT2 Inhibitors in Mouse Cardiomyocytes and Hearts: Inhibition of Na+/H+ Exchanger, Lowering of Cytosolic Na+ and Vasodilation. Diabetologia.

[72] Cefalo C.M.A., Cinti F., Moffa S., Impronta F., Sorice G.P., Mezza T., Pontecorvi A., Giaccari A. (2019). Sotagliflozin, the First Dual SGLT Inhibitor: Current Outlook and Perspectives. Cardiovasc. Diabetol..

[73] Zynquista Approved in EU for Certain Patients with Type I Diabetes. https://www.pharmatimes.com/news/zynquista_approved_in_eu_for_certain_patients_with_type_i_diabetes_1286004.

[74] Bode D., Semmler L., Wakula P., Hegemann N., Primessnig U., Beindorff N., Powell D., Dahmen R., Ruetten H., Oeing C. (2021). Dual SGLT-1 and SGLT-2 Inhibition Improves Left Atrial Dysfunction in HFpEF. Cardiovasc. Diabetol..

[75] Karg M.V., Bosch A., Kannenkeril D., Striepe K., Ott C., Schneider M.P., Boemke-Zelch F., Linz P., Nagel A.M., Titze J. (2018). SGLT-2-Inhibition with Dapagliflozin Reduces Tissue Sodium Content: A Randomised Controlled Trial. Cardiovasc. Diabetol..

[76] Schneider M.P., Raff U., Kopp C., Scheppach J.B., Toncar S., Wanner C., Schlieper G., Saritas T., Floege J., Schmid M. (2017). Skin Sodium Concentration Correlates with Left Ventricular Hypertrophy in CKD. J. Am. Soc. Nephrol..

[77] Olgar Y., Turan B. (2019). A Sodium-Glucose Cotransporter 2 (SGLT2) Inhibitor Dapagliflozin Comparison with Insulin Shows Important Effects on Zn2+-Transporters in Cardiomyocytes from Insulin-Resistant Metabolic Syndrome Rats through Inhibition of Oxidative Stress 1. Can. J. Physiol. Pharmacol..

[78] Gollmer J., Zirlik A., Bugger H. (2020). Mitochondrial Mechanisms in Diabetic Cardiomyopathy. Diabetes Metab. J..

[79] Wu C., Zhang Z., Zhang W., Liu X. (2022). Mitochondrial Dysfunction and Mitochondrial Therapies in Heart Failure. Pharmacol. Res..

[80] Depaoli M.R., Hay J.C., Graier W.F., Malli R. (2019). The Enigmatic ATP Supply of the Endoplasmic Reticulum. Biol. Rev. Camb. Philos. Soc..

[81] Cabassi A., Miragoli M. (2017). Altered Mitochondrial Metabolism and Mechanosensation in the Failing Heart: Focus on Intracellular Calcium Signaling. Int. J. Mol. Sci..

[82] Miller W.L. (2017). Disorders in the Initial Steps of Steroid Hormone Synthesis. J. Steroid Biochem. Mol. Biol..

[83] De Breda C.N.S., Davanzo G.G., Basso P.J., Saraiva Câmara N.O., Moraes-Vieira P.M.M. (2019). Mitochondria as Central Hub of the Immune System. Redox. Biol..

[84] Dadsena S., King L.E., García-Sáez A.J. (2021). Apoptosis Regulation at the Mitochondria Membrane Level. Biochimica et Biophysica Acta Biomembr..

[85] Klinge C.M. (2020). Estrogenic Control of Mitochondrial Function. Redox. Biol..

[86] Myasoedova V., Kirichenko T., Melnichenko A., Orekhova V., Ravani A., Poggio P., Sobenin I., Bobryshev Y., Orekhov A. (2016). Anti-Atherosclerotic Effects of a Phytoestrogen-Rich Herbal Preparation in Postmenopausal Women. Int. J. Mol. Sci..

[87] Popov L.-D. (2020). Mitochondrial Biogenesis: An Update. J. Cell. Mol. Med..

[88] Tilokani L., Nagashima S., Paupe V., Prudent J. (2018). Mitochondrial Dynamics: Overview of Molecular Mechanisms. Essays Biochem..

[89] Onishi M., Yamano K., Sato M., Matsuda N., Okamoto K. (2021). Molecular Mechanisms and Physiological Functions of Mitophagy. EMBO J..

[90] Chistiakov D.A., Orekhov A.N., Sobenin I.A., Bobryshev Y.V. (2014). Plasmacytoid Dendritic Cells: Development, Functions, and Role in Atherosclerotic Inflammation. Front. Physiol..

[91] Croteau D., Luptak I., Chambers J.M., Hobai I., Panagia M., Pimentel D.R., Siwik D.A., Qin F., Colucci W.S. (2021). Effects of Sodium-Glucose Linked Transporter 2 Inhibition With Ertugliflozin on Mitochondrial Function, Energetics, and Metabolic Gene Expression in the Presence and Absence of Diabetes Mellitus in Mice. JAHA.

[92] Zhou H., Wang S., Zhu P., Hu S., Chen Y., Ren J. (2018). Empagliflozin Rescues Diabetic Myocardial Microvascular Injury via AMPK-Mediated Inhibition of Mitochondrial Fission. Redox Biol..

[93] Mizuno M., Kuno A., Yano T., Miki T., Oshima H., Sato T., Nakata K., Kimura Y., Tanno M., Miura T. (2018). Empagliflozin Normalizes the Size and Number of Mitochondria and Prevents Reduction in Mitochondrial Size after Myocardial Infarction in Diabetic Hearts. Physiol. Rep..

[94] Durak A., Olgar Y., Degirmenci S., Akkus E., Tuncay E., Turan B. (2018). A SGLT2 Inhibitor Dapagliflozin Suppresses Prolonged Ventricular-Repolarization through Augmentation of Mitochondrial Function in Insulin-Resistant Metabolic Syndrome Rats. Cardiovasc. Diabetol..

[95] Shao Q., Meng L., Lee S., Tse G., Gong M., Zhang Z., Zhao J., Zhao Y., Li G., Liu T. (2019). Empagliflozin, a Sodium Glucose Co-Transporter-2 Inhibitor, Alleviates Atrial Remodeling and Improves Mitochondrial Function in High-Fat Diet/Streptozotocin-Induced Diabetic Rats. Cardiovasc. Diabetol..

[96] Lee Y.H., Kim S.H., Kang J.M., Heo J.H., Kim D.-J., Park S.H., Sung M., Kim J., Oh J., Yang D.H. (2019). Empagliflozin Attenuates Diabetic Tubulopathy by Improving Mitochondrial Fragmentation and Autophagy. Am. J. Physiol. Renal Physiol..

[97] Makrecka-Kuka M., Korzh S., Videja M., Vilks K., Cirule H., Kuka J., Dambrova M., Liepinsh E. (2020). Empagliflozin Protects Cardiac Mitochondrial Fatty Acid Metabolism in a Mouse Model of Diet-Induced Lipid Overload. Cardiovasc. Drugs Ther..

[98] Takagi S., Li J., Takagaki Y., Kitada M., Nitta K., Takasu T., Kanasaki K., Koya D. (2018). Ipragliflozin Improves Mitochondrial Abnormalities in Renal Tubules Induced by a High-Fat Diet. J. Diabetes Investig..

[99] Liu X., Xu C., Xu L., Li X., Sun H., Xue M., Li T., Yu X., Sun B., Chen L. (2020). Empagliflozin Improves Diabetic Renal Tubular Injury by Alleviating Mitochondrial Fission via AMPK/SP1/PGAM5 Pathway. Metabolism.

[100] Sa-nguanmoo P., Tanajak P., Kerdphoo S., Jaiwongkam T., Pratchayasakul W., Chattipakorn N., Chattipakorn S.C. (2017). SGLT2-Inhibitor and DPP-4 Inhibitor Improve Brain Function via Attenuating Mitochondrial Dysfunction, Insulin Resistance, Inflammation, and Apoptosis in HFD-Induced Obese Rats. Toxicol. Appl. Pharmacol..

[101] Belosludtsev K.N., Starinets V.S., Belosludtsev M.N., Mikheeva I.B., Dubinin M.V., Belosludtseva N.V. (2021). Chronic Treatment with Dapagliflozin Protects against Mitochondrial Dysfunction in the Liver of C57BL/6NCrl Mice with High-Fat Diet/Streptozotocin-Induced Diabetes Mellitus. Mitochondrion.

[102] Wang C.-Y., Chen C.-C., Lin M.-H., Su H.-T., Ho M.-Y., Yeh J.-K., Tsai M.-L., Hsieh I.-C., Wen M.-S. (2020). TLR9 Binding to Beclin 1 and Mitochondrial SIRT3 by a Sodium-Glucose Co-Transporter 2 Inhibitor Protects the Heart from Doxorubicin Toxicity. Biology.

[103] Goerg J., Sommerfeld M., Greiner B., Lauer D., Seckin Y., Kulikov A., Ivkin D., Kintscher U., Okovityi S., Kaschina E. (2021). Low-Dose Empagliflozin Improves Systolic Heart Function after Myocardial Infarction in Rats: Regulation of MMP9, NHE1, and SERCA2a. Int. J. Mol. Sci..

[104] Song Y., Huang C., Sin J., de Germano J.F., Taylor D.J.R., Thakur R., Gottlieb R.A., Mentzer R.M., Andres A.M. (2021). Attenuation of Adverse Postinfarction Left Ventricular Remodeling with Empagliflozin Enhances Mitochondria-Linked Cellular Energetics and Mitochondrial Biogenesis. Int. J. Mol. Sci..

[105] Wilson A.J., Gill E.K., Abudalo R.A., Edgar K.S., Watson C.J., Grieve D.J. (2018). Reactive Oxygen Species Signalling in the Diabetic Heart: Emerging Prospect for Therapeutic Targeting. Heart.

[106] Liu C., Ma N., Guo Z., Zhang Y., Zhang J., Yang F., Su X., Zhang G., Xiong X., Xing Y. (2022). Relevance of Mitochondrial Oxidative Stress to Arrhythmias: Innovative Concepts to Target Treatments. Pharmacol. Res..

[107] Singh H. (2021). Mitochondrial Ion Channels in Cardiac Function. Am. J. Physiol. Cell Physiol..

[108] Joiner M.A., Koval O.M., Li J., He B.J., Allamargot C., Gao Z., Luczak E.D., Hall D.D., Fink B.D., Chen B. (2012). CaMKII Determines Mitochondrial Stress Responses in Heart. Nature.

[109] Erickson J.R., Pereira L., Wang L., Han G., Ferguson A., Dao K., Copeland R.J., Despa F., Hart G.W., Ripplinger C.M. (2013). Diabetic Hyperglycaemia Activates CaMKII and Arrhythmias by O-Linked Glycosylation. Nature.

[110] Hegyi B., Borst J.M., Bailey L.R.J., Shen E.Y., Lucena A.J., Navedo M.F., Bossuyt J., Bers D.M. (2020). Hyperglycemia Regulates Cardiac K+ Channels via O-GlcNAc-CaMKII and NOX2-ROS-PKC Pathways. Basic Res. Cardiol..

[111] De Marchi U., Castelbou C., Demaurex N. (2011). Uncoupling Protein 3 (UCP3) Modulates the Activity of Sarco/Endoplasmic Reticulum Ca2+-ATPase (SERCA) by Decreasing Mitochondrial ATP Production. J. Biol. Chem..

[112] Bell D.S.H., Goncalves E. (2019). Atrial Fibrillation and Type 2 Diabetes: Prevalence, Etiology, Pathophysiology and Effect of Anti-Diabetic Therapies. Diabetes Obes. Metab..

